# A qualitative perspective of working women care providers and care receivers on eldercare: a study from India

**DOI:** 10.1186/s12877-024-04782-z

**Published:** 2024-04-16

**Authors:** Dipti Govil, Harihar Sahoo, Biswabandita Chowdhury, K. S. James

**Affiliations:** 1 International Institute for Population Sciences, Mumbai, India; 2 S.P. Mandali’s Prin. L. N. Welingkar Institute of Management Development & Research, Mumbai, India

**Keywords:** Caregiving, Care providers, Care receiver, Elderly, Working women

## Abstract

**Background:**

The paper aims to explore the elderly caregiving process in India from the perspective of both elderly as well as working women care providers, along with the challenges faced and the coping strategies adopted by them during the process.

**Methods:**

In-depth interviews with 48 participants (care providers and care receivers) from 25 multi-generational households were conducted in the slums of Mumbai and analysed using QSR-NVivo-10.

**Results:**

Working women care providers supported the needs of the dependent elderly along with performing household chores and paid work. However, the way the care was perceived and demanded, was not often same as delivered by the care providers. Care provider suffered silently with poor social, physical and emotional welling in absence of support system and lack of time. Sometimes, physically exhausted care providers unknowingly resorted to elderly abuse and neglect. At the same time, a bidirectional flow of support from elderly also existed in the form of childcare, household chores and financial support. Though caregiving overstrained the care providers, strong family ties, acknowledgement of the contributions of the elderly during their young days, and the desire to set a precedent for the young generation did not let them step back from their duties. The main coping mechanism for both care receivers and providers was largely centred around the notion of acceptance of their situation.

**Conclusion:**

Conversations between generations can help in enhancing family ties and reduce conflicts. The support of family and community can also ease the burden of caregiving.

## Background

The multi-layered process of aging entails multidimensional challenges to the society and economy and is accompanied with a high demand for support in terms of economic transfers, health expenditure, and care provisions. A staggering 19.1% of India’s population will transit into the elderly age group by 2050 from 9.4% in 2017; hence, the need for geriatric care becomes squarely important [1]. In traditional agrarian societies like India, providing care to the elderly is inherent to the culture, explained largely by the notion of filial piety [2]. In the extended family system, characterized by multi-generational co-residence, the family usually takes care (informal) of the physical, emotional and economic needs of the elderly [2, 3]. However, changes in the living arrangements and family norms are restricting the elderly from receiving adequate care due to health incapacities [4].

Caregiving is generally central to the identity of women due to their socialization into nurturing roles [5]. Hence, they are the main providers of care and support to the elderly. With the increase in educational attainment and new opportunities of work, the employment of women in various sectors has increased [6, 7]. As a result, the response to elderly care has undergone alterations [8], and caregiving has become a serious concern affecting both care receivers and care providers due to the load of multiple roles and responsibilities [8–10]. Female care providers tend to bear the burden more intensely than male members of the family due to the lower value attached to their time. Hence, it becomes important to understand how informal caregiving has shaped up in the face of persistent demand for work participation.

Increased life expectancy, along with the rising health burden due to chronic non-communicable and degenerative diseases, is leading to functional restrictions among the elderly [11]. In India, approximately 21% elderly males and 26% elderly females reportedly experience at least one ADL restriction [12]. This high burden has a debilitating effect on the individuals and their family members and making the elderly even more dependent on the others [13, 14]. As per Maslow’s hierarchy of needs framework, caregiving is one of the unmet needs of the elderly [15]. The burden of caregiving can affect a provider in multiple ways. The lost social and economic opportunity costs and reduced productivity are the prime outcomes [8]. Role overload and the resultant stress multiply the perceived burden in a household where an elderly person suffers from chronic health conditions and functional restrictions [16, 17]. Often, care providers remain physically strained and emotionally drained, which affects their wellbeing.

In India, most of the caregiving activities are centered around households rather than institutes, which makes caregiving an informal process. Despite several policy and legal norms in place, there are limited provisions for social welfare benefits and assistance for the elderly, which reinforces such inter-dependencies and makes care and support of the elderly primarily a domain of the families, especially females [18–21]. There is a paucity of research on elderly care in India. Hence, in-depth research is required to understand the aspects and process of caregiving and the burden on female care proviers. The voice of the family care providers, especially women, is a missing piece in the literature as very few studies have attempted to examine the links in light of the changing scenario. Against this backdrop, the paper aims to develop an in-depth understanding of the process of eldercare in Indian context. The process emphasizes the perspective of both elderly (care receiver) and working women care provider. The challenges faced and the coping strategies adopted by the care providers and receivers along with identifying the gaps in the elderly caregiving process have also been explored. With the rise of dual-career families and diminishing joint family system in India, the comparison of both the perspective may highlight the expectations in the context of constraints and obligations. The findings may generate evidence in the domain of eldercare within the families and direct the in-depth research to explore the dynamics further.

## Methods

### Study design

A qualitative study aiming to understand the elderly caregiving process and care provider’s burden was executed in Mumbai and its suburbs, using an interpretative phenomenological design. The design helped to capture the aspects comprehensively and included different perspectives offered by the respondents around the phenomenon of caregiving. The data was collected during the month of November 2019 from multi-generational elderly households in slum and non-slum areas. The Standards for Reporting Qualitative Research (SRQR) [22] guidelines were used for reporting this study.

### Participants and recruitment process

Interviews were conducted among elderly persons along with their care providers who were females. Households were selected based on two conditions:

1. Elderly households with working women care providers (female care providers involved in wage earning activities at the time of the survey).
2. Elderly (60 to 85 years) living with Activities of Daily Living (ADLs) difficulties like in feeding, bathing, dressing, toileting, and mobility (i.e., getting in and out of a bed or chair), suffering from incontinence (difficulty in controlling bladder and bowel movement), or having any locomotor disability.

The exclusion criteria included:

1. Elderly living with multiple family care providers.
2. Elderly relying primarily on paid care.
3. Elderly suffering from any mental health problem or those who were completely bed ridden. They were excluded as they may have had difficulty in replying during the interview.

A respondent or key informant driven sampling design/snowball sampling was used to collect the data. First, community workers were asked if there were any such households in their community. If they were aware of any elderly living with a working daughter or daughter-in-law or any other female relative, the workers were requested to introduce the interview team to such households. Though the study aimed to collect information from slum and non-slum households in the selected locations, getting information from non-slum residents was difficult. Elderly from non-slum households in Mumbai, live in gated communities, where getting permission is exteremly difficult in the wake of increasing crime. Still, researchers tried their best to contact at least a few elderly individuals living in such households. Such elderly were tracked in the local gardens and public places and were requested to participate in the study. As a result, one-third of the total sample was collected from non-slum setups and the rest from the slum population. A care provider was identified on the basis of an elderly individual’s assessment of who provided maximum care. The care providers were working women (daughters or daughters-in-law) who were involved in full-time or part-time wage-earning activities in the formal or informal sectors.

### Data collection

In-depth interviews with 48 respondents (22 women care providers and 26 elderly care receivers) from 25 multi-generational households were conducted. In three of the households, interviews with care providers could not be completed because of their absence during interview period. Though we targeted to interview at least 20 households, we went ahead with five additional households as we were not able to get sufficient interviews from non-slum background. Data saturation was achieved at 25th respondent among care receivers and 20th for care provider. Two in-depth interview guidelines (semi-structured) were prepared to cover various aspects of caregiving and care receiving, that is, care expectation, care needs, participation in household activities, challenges, care provider’s burden, and coping mechanisms along with the socio-demographic profile. The interviews were conducted mostly in the native languages, that is, Hindi and Marathi; only one respondent replied in English. Hence the interview guidelines were translated into the Hindi and Marathi languages. The face to face in-depth interviews were conducted by trained and senior staff in a private setting. One of the authors was present in all the interviews to ensure that required protocol was followed in conducting the interviews. On an average, an interview lasted for 45–60 min. The field notes taken by researchers were supplemented to the transcripts. Pre-testing of the tool was carried out before the final field work, post which some minor changes were made in the guidelines.

### Ethical consideration

The study followed all ethical consideration and the details of the same is provided in declaration section.

### Data analysis

**Fig. 1:**
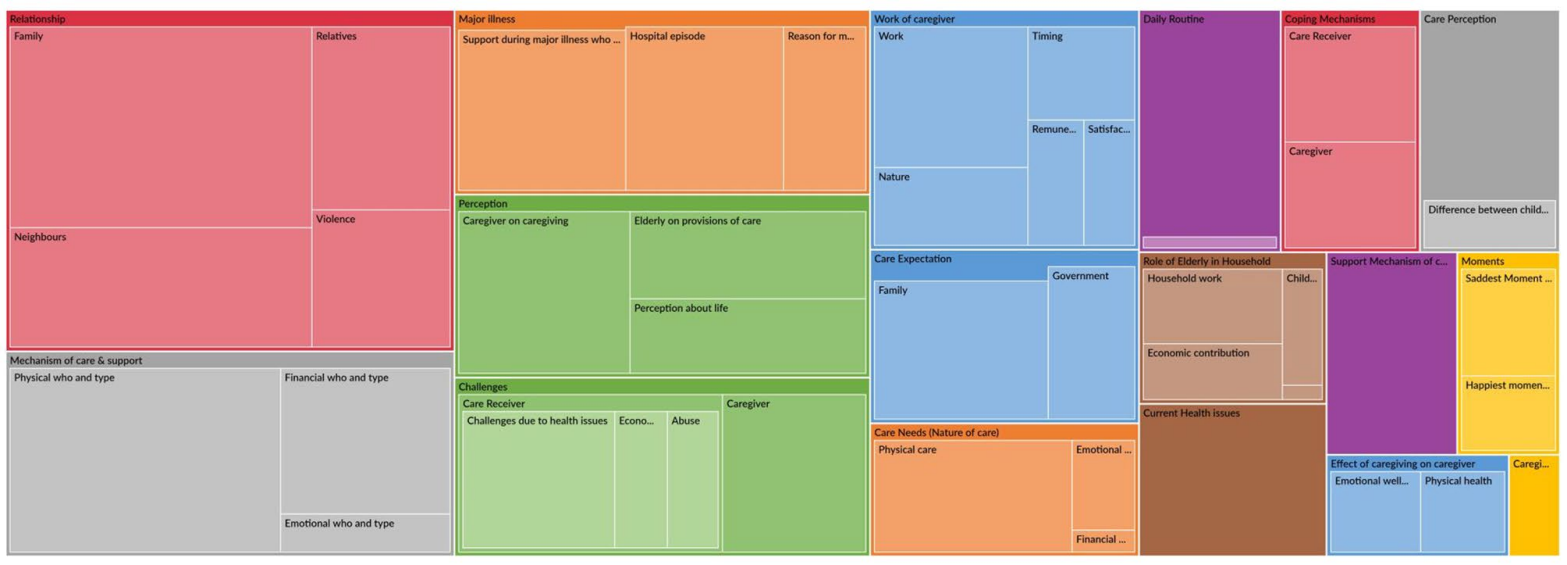
Hierarchical Chart of Coding Structure

The interviews were audio-recorded and later transcribed verbatim. Marathi transcripts were translated into the English language for better understanding of the researchers. The research team reviewed all the transcripts and translations for the accuracy. Both deductive and inductive approaches were used to analyse the data. We first identified a comprehensive list of themes (coding framework) from the interview guidelines, which was later supplemented by additional themes that emerged from the transcripts. Each transcript was read and discussed repetitively by the research team to bring out the most relevant themes and consensus. The hierarchical chart of extracted themes with coding density is provided in Fig. 1. The transcripts were analysed in QSR-NVivo 10.

The similar codes were assigned to elderly care receiver and care providers. However, the themes were not presented as a dyadic interpretation, the authors tried to bring in the perspectives of the elderly and the care providers separately and check for congruence or divergence in the themes related to caregiving process.

## Results

### Profile of the sample

More than half of the sample (55%) belonged to the Hindu religion, followed by Buddhism (23%); 46% belonged to the scheduled castes. A majority of the respondents spoke Marathi (73%). The average age of the care receivers was 73.5 years (range 60–85 years) and of care providers 40 years (range 22–54 years). On an average elderly carereceivers had 5.5 years of schooling while among careproviders, the mean years of schooling was 6.6 years. Of the total number of care receivers, 17 were females (65%) and 9 males (34%); 57.7% were widows/widowers. More than 70% of the care providers were daughters-in-law and were staying with widowed parents-in-law (60%). A majority (82%) of the care providers were currently married. Most of the elderly were financially dependent on their children and had no access to social security and had difficulty in mobility.

### Who provided care

As the elderly were living in multi-generational households, a majority of them had more than one care taker. Daughters/Daughters-in-law were the main care providers. The elderly mostly preferred to stay with their sons as per the Indian norm despite having a poor relationship with the daughters-in-law (DILs). Still DILs took care of all their basic needs. In some cases, daughters, whether married or unmarried, also provided sustained care to their frail elderly parents despite brothers being eligible to take the responsibility. In such cases, the sons seldom provided their parents monetary support or any sort of care.

A daughter who never got married and looks after her sick mother commented, “*I have a brother, but what is he even doing for my sick parents? Now-a-days, sons are different than earlier; they just think for themselves… my mother considers me as her son. I do everything for her.”* [translated].

In some cases, grandchildren, elderly spouse, and neighbours also provided care along with these two primary care providers. Grandchildren provided physical and monetary support, while spouses provided physical support such as taking the elderly to toilet, giving them a massage, and taking care of their dietary requirements.

### Care perception

In the twilight years of life, a person yearns for the same kind of care and support that they had provided to their children when they were young. In simple words, care encompasses activities or behaviours that an elderly perceives as part of support provided to them. However, it is not very clear from the previous studies as to what does care encompass exactly? In this study, an attempt was made to understand the perception of the elderly and their women care providers regarding care and caregiving. Four major themes of care emerged from the interviews: provision of food, daily chores, companionship, and respect.

#### Provision of food

The elderly felt that caring for them included providing them with ailment-specific diet characterized by various restrictions. They felt that they should be asked for their food preferences as per their taste and requirements. They highlighted that food should be given on time and it should be simple home-cooked food with little oil. These sorts of food preferences were expressed more commonly by elderly women than elderly men. Elderly men were only interested in being given food. The care providers too underscored the importance of providing suitable food to the elderly as per their health status. A 45-year-old daughter-in-law of an 85-year-old elderly man said.

*Elderly people don’t have teeth, so soft food should be served. If we have to serve them Bhakhri (Indian bread made of rice), it should be dipped in lentil soup. This makes the food soft and easy for the elderly to eat. Also, the elderly should not eat too much before going to the bed.* [translated]

#### Provision of basic necessities and help with daily chores

This issue was primarily raised by the elderly men as they were heavily dependent on women for every basic need. The elderly men felt that care should include taking care of their laundry and providing them with hot water for bathing. As many of them had mobility issues and needed support to move from one place to another, they opined that assistance in going to the washroom was essential. On their part, the care providers were concerned about the cleanliness and safety of the elderly when the latter ventured out or used a public toilet.

“*How the person is getting served is vital. Serving food, washing clothes and taking to the toilet… all these things are important. I take bath by myself but my daughter-in-law helps me to get the water. She does all the work. Each person’s help to me is important. Without help, no one can survive*,” said a 71-year-old blind elderly man who was cared by his 35-year-old daughter-in-law.

#### Companionship

Care as perceived by those who were economically dependent was restricted to basic necessities and help with accomplishing daily chores. On the contrary, for those who had a source of income, the concept of care was more intangible. Their concept of care included having a companion, having someone to listen to them, and making them feel wanted. The daughters-in-law were also of the opinion that the elderly need friends and that there should be conversations across generations. They believed that while it was important to help the elderly with the daily needs, it was equally important to make them feel happy by being by their side.

*“The elderly cannot go out alone. There is no one to talk to them. How long can they watch TV whole day? They need a friend. If there is no friend, relatives should behave like one. Then these people will feel good”*, said a 41-year-old daughter-in-law. [translated]

#### Respect

Some elderly respondents felt that care also included giving them importance and making them part of various decisions because of their age and status in the family. The elderly opined that financial stability can give them the power to negotiate their status at home and have their opinions heard; else they are just liabilities on their children. A 71-year-old man with walking difficulties expressed his feelings around this topic: 

*Old people should be somewhat financially stable so that children do not make them feel as if they are a liability*. [translated]. There was a general sentiment that their inclusion in day to day life can make them feel wanted. As a 78-year-old retired government servant explained, *“People at home and outside should at least smile at me when they see me so that I feel wanted”* [translated].

### Care needs

This section highlights the needs of the elderly, which eventually determine the care and support provided to them by their children. Broadly, the needs have been categorized as emotional, physical, and financial. The opinions of both care providers and receivers were considered.

#### Emotional care needs

Emotional needs were articulated mostly by elderly women. Many times, it was just a subtle indication about their wants and wishes, which were not mentioned to their care providers. Many of the elderly were widowed, and the younger generation had little time for them. This meant that they were haunted by loneliness. They wanted somebody with whom they could speak and share their thoughts and experiences.

“… *Sometimes I feel like talking to somebody; so in the evening I go downstairs to talk to anyone. We (the elderly) feel light by talking to others,” *said a 70-year-old widow staying with her widowed daughter. [translated]

A few of the elderly women were in the habit of waiting for their sons to come back from work and have dinner together even when their sons were abusive towards them. This way, they address the loneliness. Despite having a poor relationship with daughters-in-law, the elderly desired to keep the communication channel open to fulfil their emotional needs. “… *All I need is: somebody to ask about me; that’s it.”* said an 80-year-old widow. The elderly males did not demand such emotional care since most of them had their spouses for company. The care providers were not much aware of the emotional needs of their elderly parents/parents-in-law; thus their perspective was unavailable.

#### Physical care needs

Physical care is one of the inherent dimensions of care that needs to be addressed on a regular basis. As age progresses, the physical capabilities decline, making the elderly more and more incapable of performing the daily chores. Most of the elderly in the study required help not only to perform outdoor activities, such as travelling or meeting someone in the community, but also to perform daily activities at home, such as bathing, eating, toileting, etc., as they were suffering from various health problems. As described by a 75-year-old widow, *“… I find it difficult to use the public toilet because it is far away; but I still have to go.*” [translated].

Most of the elderly were regularly taking medicines for various morbidities. The care provider bought the medicines as well as gave them on the scheduled time. Apart from the medicines, the elderly had specific food requirements like well-blended and less oily food, less red meat, and less sugar. They required food on time and sometimes also at odd hours.

“*At 4.00 am, he wakes me up and says that he wants to have Poha or Upma and tea. Sometimes, he calls me in the middle of the night and asks for food. He cannot remain hungry because of his age,”* [translated] a 41-year-old daughter-in-law informed while talking about her father-in-law.

#### Financial care needs

 Financial car needs were not expressed by the elderly explicitly, but it was apparent through the observations and the other narratives of care receivers and care providers. It is important to mention that most of the houses were in the name of the elderly, which may have influenced how or what type of care they received and their opinions on the issue.

### Perception of care providers about caregiving

The concept of filial piety emerged from the study, whereby caring for the elderly, especially parents/parents-in-law, is considered as a duty or an obligation. Though caregiving overstrained the working women, respect for the elderly, strong family ties, acknowledgement of the contributions of the elderly during their young days, and the desire to set a precedent for their children did not let them step back from their duties. The thought that one day, they too would become old and helpless, motivated them to take care of their elderly. This sentiment was echoed by a 37-year-old daughter who was taking care of her elderly parents while juggling between marital and natal family: *My parents…, if I don’t look after them, then who will? The way we look after our parents, our children will also learn and do the same.*” [translated].

The care providers believed that the act of caregiving made them eligible to receive the blessings of their parents. They felt that the elerly deserved care as they too had provided care to their elderly in their youth. Hence, the care providers accepted the responsibility of caregiving in spite of the dual burden (work and children). While feeling empathetic towards her mother-in-law, a 54-year-old widowed daughter-in-law said, *“Out of stress, I do shout at her sometimes. But I take good care of her and provide her with food and medicines on time. I never hurt her intentionally. One day, I will also become old and experience the same situation that she is going through.”* [translated].

Many care providers considered the elderly as an asset because of their experience and wise decision-making capacities. Though the nuclear family setup is common in metropolitan cities like Mumbai, the joint family setup is considered better as it cultivates the sense of mutual care. These care providers reported to never differentiate between their parents and parents-in-law and said they provided utmost care to them. In return, they expected their parents-in-law to regard them as their daughters. The feeling of giving back also emerged in the study. Reflecting upon her past experiences, a 43-year-old daughter-in-law said, “…*The moment I got married and moved in here, leaving behind my parents, my parents-in-law started taking care of me. As a new bride, I did not know anything; my mother-in-law guided me and taught me how to cook and take care of the household……she also scolded me if I did anything wrong… When we needed their help, they were there to support us and now it is our turn to support them. Our old parents-in-law need us now. When people become old, they become like children, so we must take care of them.*.” [translated].

### Bidirectional flow of care and support

This section highlights the role of elderly in the household activities like household work, economic contribution and childcare. Caregiving is dependent upon the type of benefit received from the elderly and the nature of the relationship between the two. Contrary to the popular belief of elderly being only recipients of care and support, the elderly contributes a lot for their families in the form of taking care of the grandchildren, providing monetary support, and performing household chores. They take their grandchildren to school, bring them back from school, feed them, and spend time with them when their mother (the care provider) is at work. The elderly women even help in household work. This gives the elderly satisfaction and improves the morale of the care providers, which enables them to provide care to the elderly. We found one incidence of neglect of an elderly person since they didn’t take care of the grandchild.

The pension received by the elderly was primarily used for buying their medicines, which reduced the financial burden on the family. Their economic status allowed them to negotiate their status at home and be heard. Ownership of the house (by the elderly) helped the family financially and allowed it to save money on accommodation in an expensive city like Mumbai. This also ensured that the elderly got respect from their care providers. This was evident from a 26-year-old daughter’s-in-law statement: “W*e have a house in Mumbai, and that is because of him (late father-in-law),… we should take care of our elderly,… she (MIL) has only one son.”* [translated].

### Challenges

#### Challenges faced by care receivers

Difficulties in ambulating and the associated restrictions were the most significant challenge faced by the elderly, which subsequently affected their activities of daily living. Elderly men received help from the family to overcome the challenges; however, for a few elderly women, the challenges became a nightmare with no or limited help. Because of their immobility, they were confined to home and had restricted socialisation. The daughters-in-law were unable to spend time with the elderly outside of the time spent on cooking and helping them with the daily chores. Lack of such support was elucidated by the elderly as it had direct or indirect repercussions on them. Lack of money was a major hurdle for treatment seeking and daily needs because of no/little social security. A 66-year-old widow, living in a slum, highlighted: *I beg money from others out of compulsion. They give me 5 or 10 or15 rupees. I use it to access the toilet. Every visit costs me 10 rupees.”* [translated].

Another 66-year-old widow living in a slum said, *“I requested my daughter-in-law to get my X-ray done. It was costly; so she refused.”* [translated] There were many other similar incidences.

Elderly abuse was a crucial dimension captured by the study. Elderly women were more likely to be victims of neglect, especially by their sons. They felt helpless but due to the blood relation, they chose not to protest. As a male respondent aged 76 years said, *“He [son] does not like to talk to me; he beats me a lot… His mind is not stable; he gets mad at times. If the house is not clean, he gets furious and beats me. I don’t know why he behaves so weirdly. He also does not keep well. He has a breathing problem, that is why he gets angry”*. [translated]

#### Challenges faced by care providers

The challenges faced by the working women were related to: (a) their labour-intensive and demanding jobs that involved working as housemaids, cooks, and housekeepers and (b) to the absence of a support system for performing household activities and caregiving. They rarely get time for themselves (self-care), to pursue their dreams, engage in some entertainment, and visit friends and relatives. These challenges took a toll on their physical, emotional and social wellbeing. They neglected their physical health and experienced tiredness and body aches, which also affected their performance at the workplace.

The daily routine of the care providers leaves no room for leisure activities. Before leaving for work, they accomplish all the household chores and caregiving activities single-handedly. After returning, they make dinner, do all leftover tasks, and prepare for the grind of the next day. “*It is very much hectic to take care of an old person while doing a job. It is also hectic to do all the other things at home. We also have to manage our husbands also,”* said a 22-year-old daughter-in-law.

Some women had to forgo better job opportunities to manage and balance their caregiving and household duties. A 42-year-old never-married daughter remarked, “*I was working in a diamond cutting factory. The pay was good, but then my mother fell ill. I could not take leaves frequently… I left the job and started working as a maid*.” [translated].

Lack of support from their (drunk) husbands and long hours of engagement at work made them compromise on the wellbeing of their young children as they spent little time at home or were busy in household chores and caregiving when they were at home. They were not able to spend quality time with the children or to take care of them. A 25-year-old care provider and mother of a 5-year-old child shared, *“When my son was 2.5 years old, I used to go to the ‘dhakka’ (the port where fish is sold) for work… I used to leave my son with a neighbour and paid her money for looking after him… my husband and other family members never looked after him in his growing years.”* [translated].

Because of the overload of work at home and the workplace, the care provers experienced sleeplessness, lack of appetite, constant tiredness, back pain, body aches, fatigue, lethargy, mood swings, etc. Besides bearing the demands of their children, the mood swings of the ailing elderly increased anger, frustration, and anxiety among them. Such feelings not only impeded their emotional health, but also hampered the quality of care provided to the elderly. According to a 42-year-old daughter-in-law working as housekeeping staff, *“Managing multiple roles and responsibilities makes us feel tired and frustrated…. due to which we make mistakes and compromise our duties.”* [translated].

“*Sometimes I feel irritated by doing all this work. If I am not happy, then how are we going to help others? But our mother is like God, so we must serve her”*. [translated] (50-years-old daughter).

Many working care providers were not happy with the behaviour of their MILs and felt neglected as the latter tried to overpower and control their daughters-in-law despite the latter were actively fulfilling all the responsibilities.

### Coping mechanisms

The coping mechanism for both care seekers and care providers was largely centred around the notion of acceptance of their situation.

#### Coping mechanisms among care receivers

The elderly accepted that old age is riddled with health problems and that, therefore, they should be contented with whatever care and support they were receiving from the family. They adopted an active lifestyle (exercise/ walk) to keep themselves healthy (benefit the musculoskeletal system) and deal with health problems (prevent further functional loss).

Since elderly men did not have any responsibility related to household chores, they preferred to spend time out of their homes with their friends and neighbours in order to fight boredom and loneliness. They indulged in conversations regarding day-to-day events. They were happier to share their emotions with friends or acquaintances than with their family members. However, the study garnered mixed responses from the elderly women regarding spending time with neighbours and friends. A few elderly women went out to indulge in conversations with peers and neighbours, while others remained confined to their homes and talked to the neighbours only when the neighbours visited them.

*“I do not go anywhere, but sometimes my neighbours come to have a chat with me even though they are busy with their work,”* said a 70-year-old elderly female. [translated]

At times, the elderly did not find anybody to have a conversation as most of the neighbours were busy with their work. Hence, they resorted to talking with their grandchildren to fight loneliness and reduce stress. A 73-year-old widow said, “*I have a granddaughter. During free time, we talk to each other about what she learnt at school*… *I do not go outside; my neighbours visit me sometimes to talk to me.”* [translated].

#### Coping mechanisms among care providers

The working care providers adopted both active and passive mechanisms to cope with the situation. The working care providers had a repertoire of strategies to cope with stress, from which they chose selectively. Sometimes they accepted their situation to feel at ease, while sometimes they indulged in leisure activities to forget their work and stress for a little while. Other times, they found reliance in their family or friends and chatted their worries away. On being asked how she coped with the work, a 45-year-old daughter replied, *“…That (limited time and lot of work) is there, but what can we do? The way it is, we have to manage”*, [translated]

Another one (a 35-year-old daughter-in-law) said, “…*sometimes we talk with each other while cleaning grains… I do not find the time to go to their (neighbour) house, nor do they come to my house.”* [translated].

Many tried to avoid any arguments as they believed having arguments in the house would negatively impact their children’s and their own wellbeing. A 49-year-old daughter-in-law shared, “… *I get little time. I get bored at times, but I keep quiet and do my work. Initially, I used to feel bad, but now I just think about my daughters and don’t crib…”* [translated].

Juggling between household work, caregiving, and job, the only time they get for themselves is at night before going to bed when they watch television or browse through the mobile. But they claim that securing their financial wellbeing is more desirable than spending time on leisure or oneself.

## Discussion

This qualitative study from Mumbai and its suburban regions gathered evidence on the perception of caregiving, needs of the elderly, and challenges faced and coping mechanisms adopted by care providers (working women) and care receivers (elderly individuals). The results show that the elderly preferred to live with a child who could take care of their needs even if the child was unable to spare enough time for them. Care was not only provided by sons/daughters-in-law but also by daughters even if the elderly had a son. The married daughters sometimes had to shoulder the dual responsibility of their marital and natal home. The care providers, especially daughters-in-law, felt that they were not appreciated enough and their caregiving not acknowledged well enough despite doing everything for the elderly care receivers.

The most prominent care needs which emerged from the study included ailment-specific diet, home-cooked food with less oil, laundry, assistance in going to the washroom, and provision of hot water for bathing. Many elderly persons suffer from multiple morbidities and, therefore, require a specific diet according to their morbidities and sedimentary lifestyle. The food preferences were high among elderly women, while elderly men just wanted to get food to satisfy the hunger. In the Indian culture, since women are primarily in-charge of cooking at home, the food choices of elderly women may differ from those of elderly men. Elderly individuals who have difficulties in carrying out activities of daily living are highly dependent on their care providers for each and every type of care, which is evident from the study findings.

The literature highlights that the elderly increasingly feel that the attitude of the younger generation towards them is not desirable [23]. They seek the love, affection, and respect of the immediate family members and neighbours. The elderly in our study highlighted their need for and the importance of having someone for companionship, being listened to, and being made to feel wanted. The study highlighted that the joint family system was considered as desirable living arrangement, whereby a bidirectional flow of money and duties gave the working women the opportunity to share the responsibilities and load of caregiving. The reciprocity of relationships and transfer of resources in the joint family setup enhanced the notion of caregiving.

Working women had to compromise on their employment to look after the elderly. Having to take up low-paying jobs, seek numerous leaves without pay, or shift to other jobs which required fewer hours were the other challenges faced by the care providers. Caregiving emerged as a triple whammy for the working women as they had to manage elderly care with household chores and paid work. Physical and mental stress resulting from the burden of caregiving and other chores was a common finding throughout this study. Nevertheless, the beauty of relationships and of the Indian culture was highlighted through the concept of filial piety [2], whereby parents are considered next to GOD. This sort of support and respect was found to help the elderly experience less loneliness and have a positive state of mind.

The gender coloured role of caregiving discussed in geriatric research was manifested through the role played by the daughter/daughter in law. Co-habitation is important to manage the work and caregiving burden. Daughters/daughters-in-law adjust their time at work and house just to provide care to their near ones. This observation was supported by a study that found that women taking up high level of care or co-habitational care was not the result of their lower chance of participation in the labour force, but that it was vice-versa [24]. In a few instances, widowed mothers preferred to stay with their daughters or vice-versa. Brody and colleagues (1995) mentioned that daughters who are not currently married migrate to their natal home for co-residence with their ailing parent(s) and for economic consideration in general [25]. Satisfaction derived from caregiving is subject to the acceptance of the care provider’s role in the family. The relationship between a care provider and a care receiver is determined by their interpersonal relationship, including if the working women are appreciated for the role in caregiving. Care provision by the women, besides their household chores and paid work, is seen as an opportunity cost with a time-cost differential. Studies have argued that women manage the time dedicated to caregiving by taking up low-paid informal and flexible jobs as they can’t substitute the caregiving by any means [25].

We observed a sharp differential in the gender dimension towards care in the present study. FILs were more satisfied with their daughters-in-law as all they cared about was the fulfilment of the basic caregiving activities and didn’t interfere with the way the activities were performed. Mothers-in-law as care receivers, however, expected a lot more dedication from care provider and control over the caregiving activities. The caregiving invokes the values related to contributing to family, respecting and honouring elders, transferring those values to the younger generation, and having mutual respect.

**Fig. 2:**
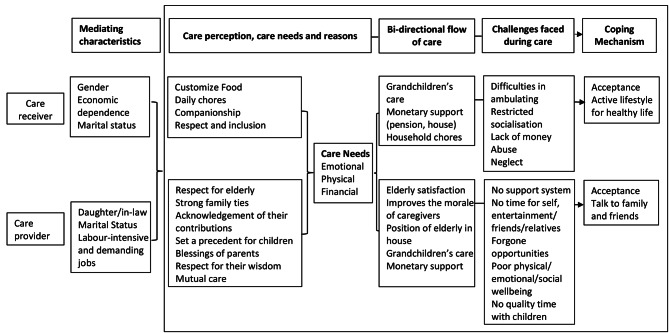
Pictorial presentation of issues emerged from narratives

The care providers in our study were engaged in paid work activity. They accomplished the physical activities related to caregiving before leaving for work. The physical support needed by the elderly for movement and the emotional support required by them due to functional restrictions on going out remained largely unfulfilled by the care providers. The overburden of work resulted in anger and frustration and neglect and abuse of the elderly. The *substitution response*, which is explained by time scarcity and caring responsibilities negatively influence the time spent at work and *discrimination effect*, which is explained by care providers as they earn less and need flexible work setups [24]. The samples in our study were mostly of those residing in slums from the lower socio-economic background. Earning was not a choice but a compulsion for the care providers to maintain the livelihood of their families. The burden of household chores, caregiving, and paid work limited their mobility in occupation, caused them physical-emotional stress, and affected their care towards their children. However, it was noticed that they also accepted the challenge positively. In certain instances, the vested interest of acquiring property from their parents-in-law motivated the care providers to regard the duty of caregiving as fruitful and demonstrate a sense of dedication. At the same time, there were women care providers who believed that the elderly deserved care, principally due to the notion of filial care, which led them to dedicate their time and effort at their natal or marital home. The pictorial presentation of the issues emerged from the study are presented in Fig. 2.

The study captured different aspects of elderly care and the role of working women as care providers in a comprehensive way. It could not, however, capture the nature of illness and the associated care (exclusive) required by the elderly. A scale that measures the extent of functional restriction could give deeper insights into the demand for care. A look into the number of hours put in by the care providers would be helpful in analysing the priority attached to work and caregiving in reference to other activities. The study constituted a socio-economically backward sample majorly from the slum settlements. A bigger sample covering the elderly and their families from non-slum and gated communities with a higher socio-economic background would reveal the bigger picture. This suggests a need for an in-depth study focusing on the other aspects of health, socio-economic conditions, and caregiving demands of the elderly in wider setups.

## Conclusions

The study aimed to capture the nature of care relationship between elderly individuals and women care providers in the households. The way care is perceived and demanded by the elderly is often not the same as delivered by the women, given that they were engage in paid work in order to support their families and are burdened with several responsibilities all of which they have to accomplish on their own. Dependency and emotional dissatisfaction among the elderly were observed in the study. At the same time, the burden of caregiving and physical and emotional stress were found to affect the life satisfaction of the working care providers. Women had to rely upon their neighbours or grandchildren for the support of the lonely elderly in their absence due to work. The role of the community in the informal care market was realized in terms of the social capital the community possess among its members. Therefore, the role of civic groups at the community level needs to be highlighted as they have significant potential to take care of the aging population in the future.

The needs of the elderly are many and complex, but their physical and mental health needs predominate given their fragile health. Though the relationship among family members was good, the physically exhausted and stressed care providers unknowingly resorted to elderly abuse and neglect. In such a scenario, support from the family and co-operation of the elderly would not only help in alleviating the care provider’s work pressure but would also relieve their sense of burden. Though the elderly were contented with the care and support they received, they were confined indoors and could not attend social events, visit religious places, or travel without support. Financial assistance from the government in the form of pension and healthcare assistance is a must to reduce the burden of care.

## Data Availability

The datasets used and/or analysed during the current study are available from the corresponding author on reasonable request.
